# Comparison of Amplitude Measurements on Borehole Geophone and DAS Data

**DOI:** 10.3390/s22239510

**Published:** 2022-12-05

**Authors:** Sana Zulic, Evgenii Sidenko, Alexey Yurikov, Konstantin Tertyshnikov, Andrej Bona, Roman Pevzner

**Affiliations:** Centre for Exploration Geophysics, Curtin University, Bentley, WA 6151, Australia

**Keywords:** borehole seismic, geophones, DAS, innovative technologies

## Abstract

DAS and geophones are the two most popular sensors for borehole seismic acquisition. As such, it is important to get a good understanding of how these two types of sensors compare to each other. The natural measurand for the techniques is different; millivolts are approximately proportional to particle velocities for geophones vs. changes in the phase of light linked to the changes in strain on the sensing fibre. This paper focuses on the experimental comparison of absolute values of these measurands derived from a VSP survey acquired in Curtin GeoLab training well. We describe the acquisition setup for the walk-away VSP acquired with DAS and geophones, allowing the direct comparison and the workflow, which we can use to represent the data in strain rate. Albeit this is unlikely to be universal, we find that the absolute values are similar for this experimental setup.

## 1. Introduction

The rapid uptake of distributed acoustic sensing (DAS) for borehole seismic acquisition prompts fundamental research to identify how its performance compares to the performance of conventional borehole geophone sensors. These two types of sensors, DAS and geophones, are fundamentally different. The geophones are electro-mechanical sensors that sense the motion of the earth in the form of particle velocity. In contrast, DAS observes a change in elongation of an optical fibre that acts as the sensor. The geophone consists of a permanent magnet mounted on a spring with a conductor (copper wire or coil) around it. When the earth moves, the magnet and coil also move. This movement generates a voltage across the coil’s winding, which is proportional to the rate at which the coil cuts the magnetic flux, that is, to the velocity of the earth’s motion [1]. In the case of borehole geophone, the geophone is mounted inside a downhole probe’s housing, suspended in the borehole by an armoured cable and clamped to the formation at each desired depth. DAS utilises a fibre-optic (FO) cable, along which the interrogator unit (IU) emits a light pulse and registers how the phase of the backscattered light changes over time in sampling points [2]. The change in phase of backscattered light is proportional to the fibre deformation caused by the earth’s motion. Although there is no standard for a DAS system in terms of its generic architecture, in summary, DAS measurements are said to be proportional to the average strain or strain rate between two sampling points separated by a gauge length.

For borehole seismic measurements, a fibre-optic sensing cable is deployed in the well. Borehole geophones and DAS have different factors that affect the measured signal. For example, the geophone measurements are affected by the geophone type and its performance, the probe’s housing, the quality of the probe’s coupling to the formation or the casing, etc. [3,4]. The DAS measurements are affected by the cable design, the cable’s coupling to the formation, optical parameters, the interrogator design, etc. [2,5,6,7]. Therefore, it is reasonable to question whether these systems can obtain the same information about the same physical properties. Wang, et al. [8] demonstrated on real and synthetic data that amplitudes of the strain waveforms computed from geophone waveforms are comparable to those obtained by DAS. 

Initial DAS tests for borehole seismic application used preinstalled cables behind the casing or on tubing inside the casing and demonstrated that seismic energy could be detected on FO cable at a depth over 4 km [9,10,11]. Daley, et al. [12] showed a variety of DAS deployment and acquisition possibilities, in which data quality varied significantly depending on these. Mateeva, et al. [5] qualitatively compared geophone to DAS and concluded that DAS polarity is independent of the wave propagation direction and that while transmitted waves have the same polarity as on the geophone dataset, the reflected DAS waves have opposite polarity to reflections on the geophone dataset. Dean, et al. [13] attempt to clarify the nature of DAS measurements, and on examples from synthetic and real data, they show their DAS system measure instantaneous strain and that the time derivative of strain (the strain rate) is proportional to the average velocity measured at all points along the gauge length. In addition to this, they showed that DAS has highly complex directionality that is dependable on wavenumber and angle of incidence. Correa, et al. [6] compared seismic data obtained by DAS and conventional borehole seismic sensors to understand DAS data more and showed that DAS could provide similar or better data quality. As DAS measures strain or strain rate, and geophone measures particle velocity, several methods could be used to convert one property to the other. Most of the published literature is related to the conversion of DAS data to particle velocity, where it is either necessary to rescale the time-integrated DAS data [14] or apply a conversion filter [15] which accounts for pulse and gauge length. The former requires the knowledge of local propagation velocity, while the latter needs to be regularised by adding the noise to DAS data to avoid division by zero that may occur for certain wavenumbers. However, Isaenkov, et al. [16] had difficulties applying the filter for larger wavenumber and suggested that internal interrogator processing may contribute to DAS response deviating from a strain rate.

Moreover, various DAS interrogators can have very different designs. The differences in the design of DAS recording units and selection of acquisition parameters significantly affect the data quality [2,13,17,18]. Therefore, further research on DAS is required to better identify the strengths and limitations of DAS technology and assess how DAS measurements correspond to standard borehole geophone recordings.

These tasks are best accomplished in a controlled environment at designated test sites that should allow for the repetition of the experiment using different sensors in stable conditions. In addition, such test sites should enable the comparison of different seismic receivers for both borehole and surface arrays using different types of seismic sources. There are several test facilities for carbon capture and storage (CCS) around the globe, where DAS technology is used, including CaMI [19], Otway International Test Centre [20], CSIRO In-Situ Laboratory [21] and RITE [22]. However, access to the data from these project-based sites is often restricted and not publicly available. Furthermore, this type of testing is not limited to these CCS sites; standard telecommunication fibre-optic (FO) cables as seismic DAS sensors were compared to conventional seismometers by Lindsey, et al. [23], Lindsey, et al. [24] and Ajo-Franklin, et al. [25]. Lastly, the fundamental research on DAS is conducted at specifically developed test sites for trialling DAS technology: Aramco Houston Research Centre built for testing borehole arrays [26]; NOR-FROST [27] used to study the performance of surface DAS arrays and their coupling in different mediums; and the GeoLab Research facility at Curtin University, where DAS response can be studied from both surface and borehole DAS array, and compared to the reference geophone datasets. 

In this paper, we compare DAS and geophone data acquired during the walkway vertical seismic profile (VSP) experiment performed at the Curtin GeoLab in 2020. First, we convert the particle velocity data from the geophone dataset to the strain rate. Then we use the first-break amplitudes and the entire wavefield to compare the absolute values of the strain rate of both types of sensors. We demonstrate that the DAS response is similar to the geophone response converted to the strain rate averaged over the gauge length of the DAS system. Furthermore, we show that the amplitudes of the strain rate measured with DAS have similar absolute values to strain-rate amplitudes obtained using converted geophone measurements—the losses of seismic energy due to the geophone coupling and housing are similar to the losses in the fibre-optic cable construction and installation.

## 2. Test Site: Curtin GeoLab Research Facility

The Curtin GeoLab Research Facility was established at Curtin University, Western Australia, with aims to conduct applied geophysical research, equipment testing and training of students and industry personnel. The key components of the GeoLab are a 900 m vertical well drilled on Curtin campus and state-of-the-art seismic equipment, which includes surface and borehole three-component geophones, impulse and vibroseis seismic sources and a variety of fibre-optic cables (including a cable permanently deployed in the borehole). 

**Figure 1:**
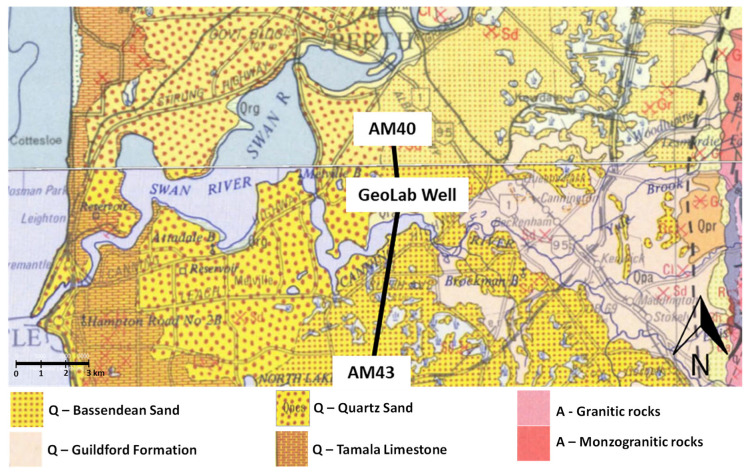
Geological map of the area around GeoLab Well-01 (© State of Western Australia (Department of Mines, Industry Regulation and Safety, 2020).

**Figure 2:**
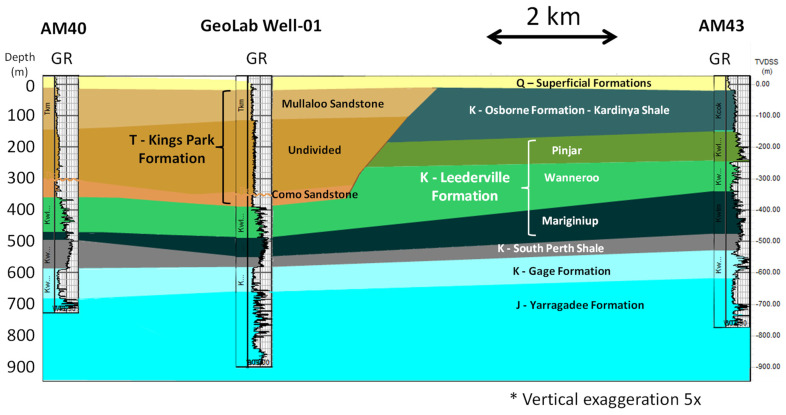
Geological cross-section along with three selected wells: AM40, GeoLab Well-01 and AM43.

The Geolab Well (NGLd), previously referenced as the NGL training well [28], is located in the eastern onshore margin of Perth Basin (Figure 1). It comprises Quaternary (Q), Tertiary (T), Cretaceous (K) and Jurassic (J) aged sediments (Figure 2). The well is cased with fibreglass slotted between 650 and 890 m depth. A fibre-optic cable is cemented behind the casing with the loop at the bottom. The cable contains two single-mode fibres for distributed acoustic sensing and two multimode fibres for distributed temperature sensing. Since the entire length of the well is covered with fibre cores four times, this facility allows multiple FO interrogators to be used simultaneously [17]. It is also possible to deploy FO cables directly into the well to test the performance of different types of cables and the effect of the coupling on the measurements.

The GeoLab facility was previously used for a wide range of geophysical experiments: some were dedicated to comparing different borehole seismic receivers [28], and other experiments utilised different interrogator units and studied their internal characteristics and performance of various cable designs [18]. Shulakova, et al. [29] examined the use of the GeoLab well for passive registration of earthquakes and technogenic activities using DAS.

**Figure 3:**
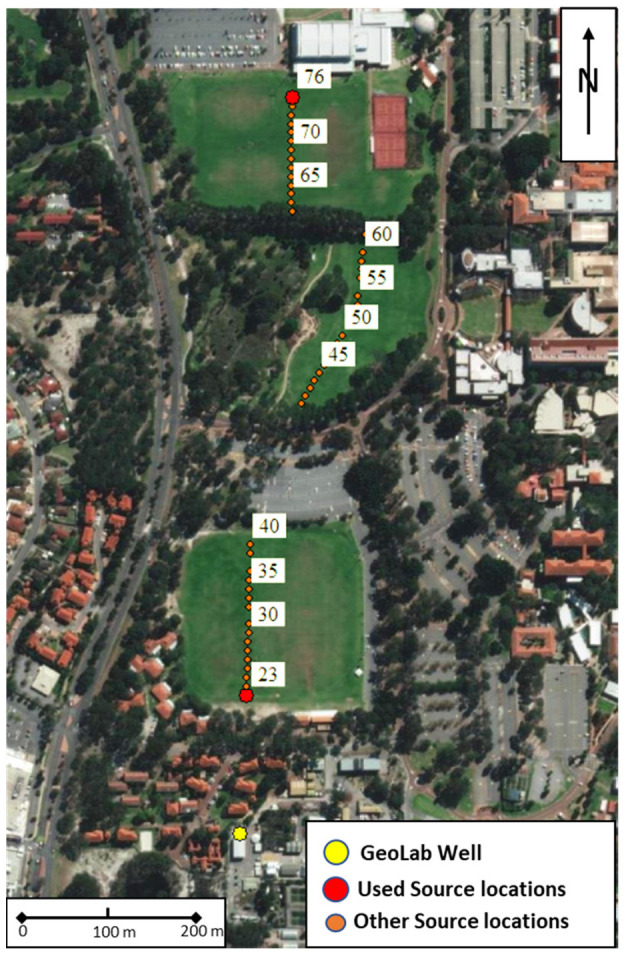
Aerial map showing the surface location of the well (blue) and source positions (red); every fifth source station is labelled.

This study uses data acquired during the walk-away VSP experiment carried out at the GeoLab in June 2020. The survey area with all source locations is shown in Figure 3. Aerial map showing the surface location of the well (blue) and source positions (red); every fifth source station is labelled. The data were acquired with a conventional three-component borehole geophone and a single-mode straight FO cable cemented behind the casing of the GeoLab Well-01. The seismic signal was generated by a 26,000 lb UniVib vibroseis truck. Acquisition parameters are given in Table 1.

## 3. Method

Direct comparison of geophone and DAS performance requires converting the data to the same physical property. In practice, DAS response is often converted to the particle velocity for comparison with geophone data [6,14]. Bakku [30] indicated that when we assume that DAS measures the strain rate, then it is given by the difference in the particle velocities measured by two vertical component geophones separated by a gauge length L:

(1) $${\dot{\varepsilon }}_{zz}^{DAS}=\frac{\upsilon_{z}\left(z+\frac{L}{2}\right)-\upsilon_{z}\left(z-\frac{L}{2\;}\right)}{L},$$

where ${\dot{\varepsilon }}_{zz}^{DAS}\;$is the strain rate along the vertical direction measured by a DAS system, $\upsilon_{z}$ is the vertical component of particle velocity and z is the depth. We use this approach to convert the vertical component of particle velocity to the vertical component of strain rate.

We apply the following processing flow to get an absolute strain rate from the geophone dataset (summarised in Table 2). After loading raw geophone data, we apply descaling and geophone factors to calibrate the native system units (mV) to particle velocity (m/s). The conversion is based on acquisition parameters such as sampling rate, acquisition gain and the geophone type. The respective factors are obtained from the manufacturer’s manual. Then we differentiate data over the 10 m interval (same interval as the gauge length used during acquisition of the DAS data) and assign the field geometry. The output dataset represents the strain rate (nanostrain/s), and we refer to it as converted geophone data. DAS native measurements are the phase variation of the laser light over time. Its native measurand is radians per second and is calibrated to the absolute strain rate (nanostrain/s) based on the acquisition settings of the gauge length, laser light wavelength and sampling frequency. The corresponding descaling factor provided by the manufacturer was applied. Then the geometry is assigned. 

We firstly visually compare gathers from geophone, DAS and converted geophone datasets. Then we compare the amplitude spectrum of the entire absolute strain-rate wavefield (uncorrelated) and the first-break amplitudes (correlated) for DAS and converted geophone. 

The gauge length is one of the most important DAS parameters as it affects both the resolution and signal-to-noise ratio of the acoustic signal [13]. The IU used in this survey has a fixed gauge length, and therefore, it was not possible to study the effect of the gauge length change. The proposed conversion allows adjustment of the gauge length. We can simulate different gauge lengths by differentiating the geophone data over different depth intervals. This simulation can assist with understanding this parameter and potentially use it for the sensitivity optimisation for a target wavelength. In addition to amplitude spectrum analysis and differentiation over a 10 m interval, we simulate the DAS response with two additional gauge lengths of 50 m and 90 m.

## 4. Data Analysis

To compare the performance of the DAS system and geophones, we use data from two source locations at the near offset (source location 23, 160 m) and the far offset (source location 76, 845 m).

**Figure 4:**
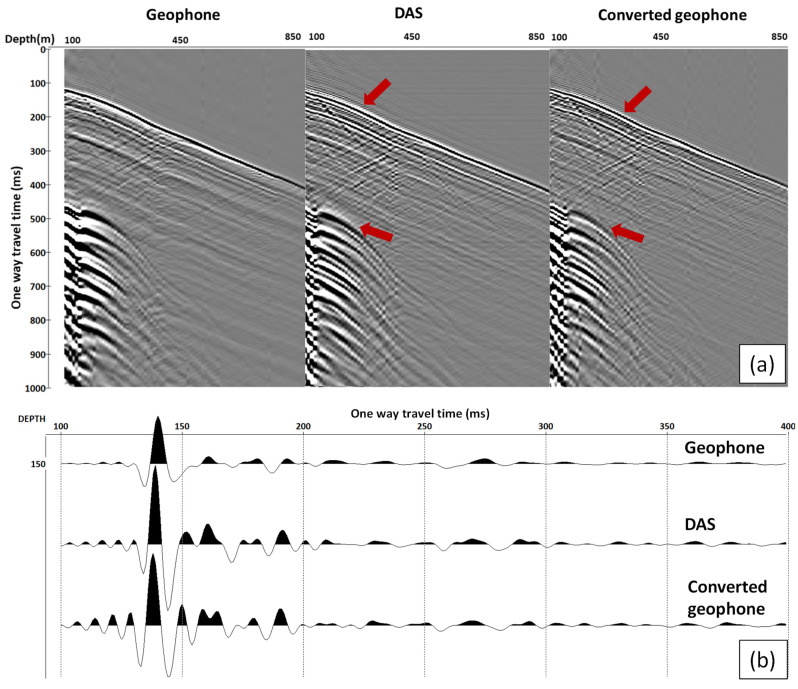
Near-offset example: (**a**) from left to right: gathers of geophone, DAS and converted geophone response; (**b**) from top to bottom: trace at a depth of 150 m of geophone, DAS and converted geophone response. We exclude every second trace from DAS data to match the same receiver interval for geophone/converted Geophone and DAS. The red arrows indicate the difference in phase of P- and S direct waves between recorded DAS and converted geophone data.

**Figure 5:**
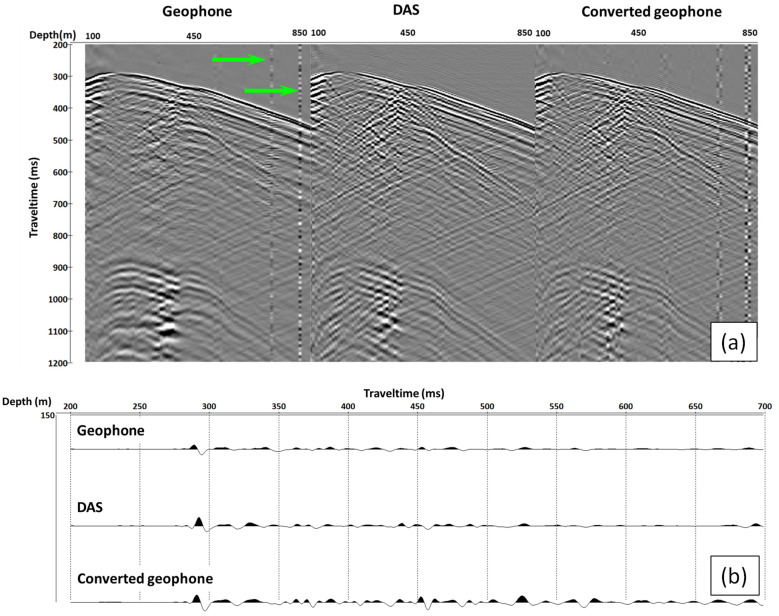
Far offset example: (**a**) gathers of geophone, DAS and converted geophone response; (**b**) trace at a depth of 150 m of geophone, DAS and converted geophone response. We exclude every second trace from DAS data to match the same receiver interval for geophone/converted geophone and DAS. The green arrows indicate locations of malfunctioning geophones.

Figure 4 and Figure 5 show the comparison of the correlated vertical component geophone, DAS and converted geophone datasets for near and far offset, respectively. The geophone data have noisy channels because of the malfunctioning of two geophones in the receiver string (green arrows in Figure 5a), which is more prominent in the far offset data as the amplitude of the signal decays. This type of noise is consequently carried through to the converted geophone dataset. Apart from that, DAS and converted geophone records look similar, as shown in the selected trace view displayed at the bottom of the figures. The red arrows indicate the difference in phase of P- and S direct waves between recorded DAS and converted geophone data.

**Figure 6:**
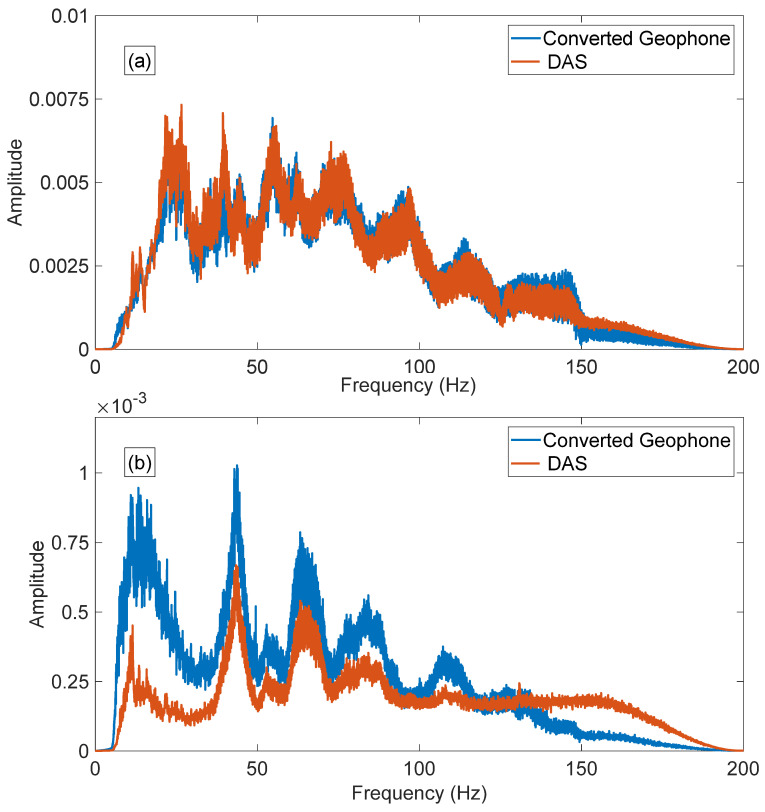
Amplitude spectrum comparisons for near (**a**) and far (**b**) offsets. The blue line is the strain rate from converted geophone, and the red line is the strain rate from DAS.

To compare the absolute values of the strain rate obtained using both types of sensors we analysed the uncorrelated wavefield. First, we applied Ormsby bandpass filter 5–8–150–200 Hz to denoise the data and adjusted the length of uncorrelated datasets to 27 s. Then we computed the average amplitude spectrum of the entire seismogram. Figure 6 shows the amplitude spectrum for near (a) and far (b) offsets for both types of receivers. 

The frequency spectrum of DAS and converted geophone data are similar for the near-offset shot. However, we observe more discrepancy in the spectrum with increased offset. The low-frequency signal of the DAS data has a lower amplitude compared to the converted geophone data, and towards the high frequency, the entire spectrum is biased (Figure 6b). Discrepancies for far offset data are most likely to be attributed to different directional sensitivity of DAS and geophones as at such offsets, seismic waves approach the vertical well at quite large angles. The prominent spikes/notches on the far offset spectra are the results of the interference of the multiples. 

**Figure 7:**
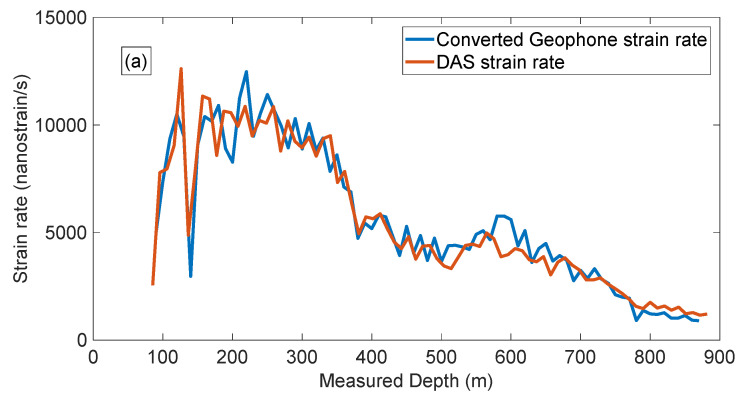

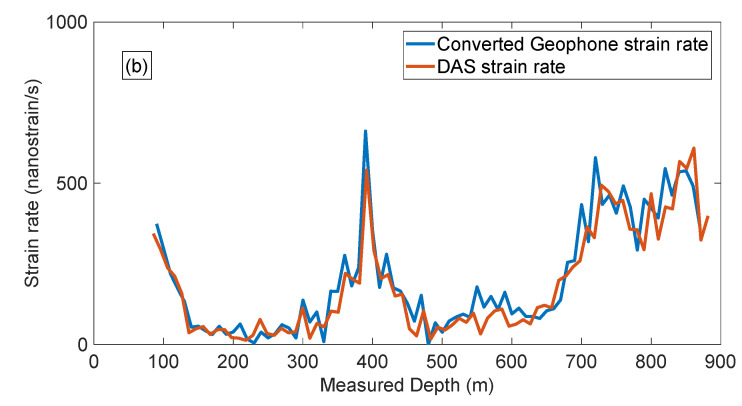
First-break amplitude comparison for near (**a**) and far (**b**) offset. The blue line is the strain rate from converted geophone, and the red line is the strain rate from DAS.

Then we correlate the data with the pilot sweep and compare the first-break amplitudes for near and far offsets (Figure 7a,b, respectively).

**Figure 8:**
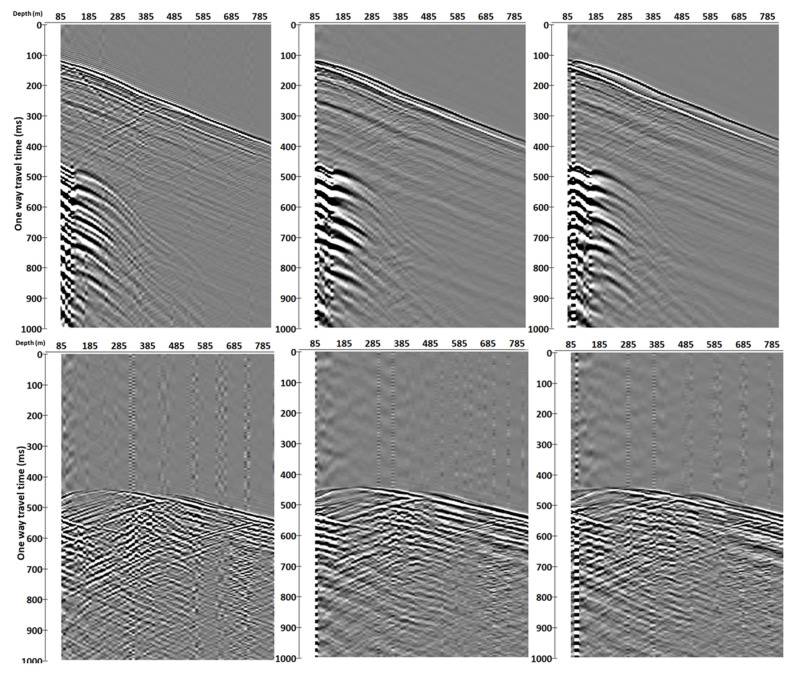
Gauge length simulation of 10 m (**left**), 50 m (**middle**) and 90 m (**right**), for near (**top**) and far (**bottom**) offset converted geophone.

In addition to amplitude spectrum analysis, we simulate the DAS response with two additional gauge lengths by differentiation of geophone data over different depth intervals. The examples of converted geophone datasets obtained by differentiation over three simulated gauge lengths are shown in Figure 8, where the simulated gauge lengths are 10 m, 50 m and 90 m, from left to right.

## 5. Discussion

Our analysis shows that the conversion of geophone data (vertical particle velocity) to vertical strain rate brings the phase of the direct wave closer to the phase of DAS data (Figure 4 and Figure 5). The polarity of the reflected waves in the converted geophone dataset also matches the DAS data. The amplitude response of the studied borehole geophones and DAS is similar after converting both datasets to strain rates (Figure 6 and Figure 7). This is a surprising result considering that different factors affect geophones and DAS measurements. For example, the geophone amplitudes are affected by the type of a geophone and its performance, the probe’s housing and the quality of the probe’s coupling to the formation or the casing. The DAS amplitudes are affected by the designs of a cable and an interrogator, the cable’s coupling with the formation and selected optical parameters. Nonetheless, the amplitude spectra of the two datasets show similar absolute values (Figure 6), and the variations of the first-break amplitudes with depth are similar as well (Figure 7).

Apart from the higher noise level on geophone data observed for far offset, DAS and converted geophone records look similar. However, near-offset data with higher frequencies present in the record show a significant discrepancy in the phase of the direct arrivals (arrows in Figure 4). The discrepancy is better observed at shear waves, which could be caused by how the fibre reacts to S-waves. While we can assume that the effect linked to the gauge length dominates for relatively low frequencies, high frequencies may require pulse length effect also being taken into account.

For the same source effort (single shot), DAS and converted geophone amplitudes are similar for near offset. However, for far offset, the high-frequency part of the DAS spectrum (>100 Hz) looks constant even beyond the frequency of the sweep (150 Hz), which might indicate that this part of the spectrum is mostly noise (Figure 6). This agrees with earlier findings that the geophone has higher sensitivity with increased offset than DAS. 

**Figure 9:**
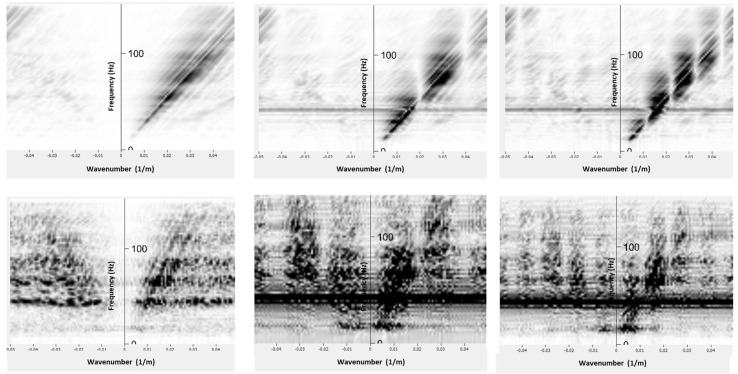
F-K spectrum for the converted geophone data with a simulated gauge length of 10 m (**left**), 50 m (**middle**) and 90 m (**right**) for near (**top**) and far (**bottom**) offset.

Similar strain-rate response between DAS and converted geophone datasets suggests using dense geophone data to simulate DAS measurements. Simulation of DAS response allows analysis of strain-rate wavefields and further understanding of DAS data (e.g., adaptation of existing particle velocity processing workflows to DAS workflows). For example, the gauge length is one of the most important parameters for DAS survey [13] as it affects not only the signal-to-noise ratio but also the wavefield distortion. Changing the gauge length can Introduce a ghost wave if the gauge length is not optimal for the recorded wavelength (Figure 8). In addition to this, we can observe the notches in k-spectrum, which are caused by the gauge length effect (Figure 9). In the absence of standardisation of DAS systems (i.e., different DAS systems may have fixed or variable gauge length parametrisation), one can study the gauge length effect from the converted geophone wavefield and potentially use it for the sensitivity optimisation for a target wavelength. Having data with densely spaced geophones allows one to simulate and study the effect of various gauge lengths by differentiating it on different bases.

For purposes of comparing the same physical property collected with a different type of sensor, the conversion approach by differentiation of particle velocity over the gauge length could be better than the integration of strain proposed by Bona, et al. [15] because the division with zero (for certain values of k) is avoided. 

The first-break strain-rate amplitudes are calculated from the vibroseis data correlated with a pilot sweep. This process affects the phase of a signal. The vibrator output is defined in terms of its baseplate motion (e.g., displacement, velocity or acceleration). When correlation with a pilot sweep is performed on motion data, the resulting correlogram is assumed to be zero-phase [31]. Therefore, correlating strain data with a pilot sweep (representation of motion) may impact a phase of correlated DAS data. In addition to this, the effects of DAS parameters (pulse and gauge length) are shown to have a considerable effect on phase [15]. This needs to be studied further and considered when interpreting the DAS data.

## 6. Conclusions

We brought geophone and DAS measurements to the same property and calibrated them to the absolute strain rate. We compared the amplitude spectrum of the entire absolute strain-rate wavefield (uncorrelated) and the first-break amplitudes (correlated) for DAS and converted geophone. In addition to amplitude spectrum analysis, we simulated the DAS response with two additional gauge lengths.

We observed that DAS and geophone have similar absolute values of strain-rate amplitudes, despite different factors affecting the performance. The losses of seismic energy due to the geophone coupling and housing are similar to the losses in fibre-optic cable construction and installation. Albeit similarity in absolute response is surprisingly good, we do not expect this finding will be universal, different cable design, coupling, etc., can produce situations where geophone and DAS response can be different. 

A denser geophone dataset can be used to simulate DAS response.

GeoLab provided an excellent opportunity for such kind of research. Different equipment can be tested and assessed versus the reference dataset. Data is publicly available.

## Figures and Tables

**Table 1:** Seismic acquisition parameters.

VSP Survey Information	
**Geophone Recording System**	WaveLab II
**Software acquisition**	WaveControl
**Logging cable**	2500 m 4 conductor cable + electric winch
**Geophone Information**	
**Downhole tool**	Sercel 3C SlimWave with ten downhole shuttles Sensor: Omni 2400 15 Hz
**Receiver step**	10 m
**Sweep + listen time**	24 s + 4 s
**Sample rate**	1 ms
**Sweep per receiver**	1
**DAS Information**	
**Interrogator unit**	Silixa iDAS v2
**Fibre-optic cable**	Single-mode straight loose tube cable
**Sampling interval raw/binned**	1 m/5 m
**Pulse length**	5 m
**Gauge length**	10 m
**Pulse Repetition Frequency**	16 kHz (downsampled to 1 kHz)
**Sample rate**	1 ms (decimated to 2 ms for analysis)
**Sweep per receiver**	9
**Source Information**	
**Source type**	26,000 lb UniVib
**Source sweep**	8–150 Hz linear sweep
**Sweep duration**	24 s
**Tapers**	0.5 s
**Force**	70%
**Source control**	Pelton VibPro
**Number of source locations**	48

**Table 2:** Processing workflow for getting the absolute strain rate from geophone and DAS measurements.

Step	Converted Geophone	DAS
1	Read raw SEG-Y	Read relative strain-rate iDAS data
2	Apply descaling factor to get the voltage in millivolts	Apply descaling factor to get absolute strain rate
3	Apply geophone factor to get particle displacement rate in m/s	Assigning geometry
4	Assigning geometry	
5	Differentiate over the GL interval	

## Data Availability

The baseline geophone dataset and corresponding DAS dataset are available at Research Data Australia (https://doi.org/10.25917/7h0e-d392).
